# Cilostazol Inhibits Accumulation of Triglyceride in Aorta and Platelet Aggregation in Cholesterol-Fed Rabbits

**DOI:** 10.1371/journal.pone.0039374

**Published:** 2012-06-22

**Authors:** Hideki Ito, Kenji Uehara, Yutaka Matsumoto, Ayako Hashimoto, Chifumi Nagano, Manabu Niimi, Goro Miyakoda, Keisuke Nagano

**Affiliations:** 1 First Institute of New Drug Discovery, Otsuka Pharmaceutical Co., Ltd., Tokushima, Japan; 2 Free Radical Research Project, Otsuka Pharmaceutical Co., Ltd., Tokushima, Japan; Institut National de la Santé et de la Recherche Médicale, France

## Abstract

Cilostazol is clinically used for the treatment of ischemic symptoms in patients with chronic peripheral arterial obstruction and for the secondary prevention of brain infarction. Recently, it has been reported that cilostazol has preventive effects on atherogenesis and decreased serum triglyceride in rodent models. There are, however, few reports on the evaluation of cilostazol using atherosclerotic rabbits, which have similar lipid metabolism to humans, and are used for investigating the lipid content in aorta and platelet aggregation under conditions of hyperlipidemia. Therefore, we evaluated the effect of cilostazol on the atherosclerosis and platelet aggregation in rabbits fed a normal diet or a cholesterol-containing diet supplemented with or without cilostazol. We evaluated the effects of cilostazol on the atherogenesis by measuring serum and aortic lipid content, and the lesion area after a 10-week treatment and the effect on platelet aggregation after 1- and 10-week treatment. From the lipid analyses, cilostazol significantly reduced the total cholesterol, triglyceride and phospholipids in serum, and moreover, the triglyceride content in the atherosclerotic aorta. Cilostazol significantly reduced the intimal atherosclerotic area. Platelet aggregation was enhanced in cholesterol-fed rabbits. Cilostazol significantly inhibited the platelet aggregation in rabbits fed both a normal diet and a high cholesterol diet. Cilostazol showed anti-atherosclerotic and anti-platelet effects in cholesterol-fed rabbits possibly due to the improvement of lipid metabolism and the attenuation of platelet activation. The results suggest that cilostazol is useful for prevention and treatment of atherothrombotic diseases with the lipid abnormalities.

## Introduction

According to the response-to-injury hypothesis, endothelial dysfunction triggers atherosclerosis progression [1]. Platelets are activated and aggregated on the exposed subendothelial tissues when the endothelium is wounded in the atherosclerotic lesion. Atherosclerosis also progresses with an elevation of serum cholesterol, and platelets have been shown to be activated in patients and rabbits with hypercholesterolemia [2]–[3]. If the hypercholesterolemia continues for a long period, atherosclerosis progresses in major arteries, and eventually may result in cardiovascular diseases. Since platelets play a pivotal role in atherosclerosis progression, they represent a key target for anti-atherothrombotic therapy [4].

For the evaluation of platelet function in atherothrombotic diseases, platelet aggregation can be measured. However, the conventional light transmission method [5] is not suitable for the measurement of platelet aggregation under a hyperlipidemic condition because the chylous plasma hampers light transmission. As an alternative method, platelet aggregation can be measured using whole blood with impedance or screen filtration pressure (SFP) [6]–[7]. The SFP method has been demonstrated to be reproducible in addition to being easy to handle [8].

Cilostazol, a selective inhibitor of phosphodiesterase 3, is a vasodilating anti-platelet drug [9]. It has been used for the treatment of ischemic symptoms in patients with chronic peripheral arterial obstruction and for the secondary prevention of brain infarction [9]–[10]. In experimental studies, cilostazol has been shown to prevent thrombus formation indicating its potent anti-thrombotic effect based on its inhibitory action towards platelet function [11]–[12]. Recently, it has been reported that cilostazol attenuated atherosclerosis in low density lipoprotein receptor knock out mice and Apo-E knock out mice [13]–[15]. Some reports indicated that cilostazol improved lipid metabolism. For example, cilostazol decreased serum triglyceride and increased the serum high-density lipoprotein cholesterol (HDL-C) in mice [13]–[15], rats [16]–[17] and human [18]–[22]. In the present study, we analyzed for the first time, the serum lipoprotein from rabbits treated with cilostazol using high-performance liquid chromatography (HPLC).

Transgenic mice and rabbits have been extensively used as atherosclerosis-prone experimental animals. However, mice and rats are naturally deficient in cholesteryl ester transfer protein activity, unlike humans and rabbits. It is known that New Zealand White rabbits have low plasma total cholesterol concentrations, high cholesteryl ester transfer protein activity, low hepatic lipase activity, and lack an analogue of human apolipoprotein A-II, providing a unique system in which to assess the effects of human transgenes on plasma lipoproteins and atherosclerosis susceptibility [23]–[24]. Additionally, as the rabbits become hyperlipidemic by eating a high fat diet, it makes them an appropriate model to assess the effects of drugs for their potential use in the treatment of dyslipidemia.

In the present study, we evaluated whether cilostazol prevents atherogenesis and platelet aggregation in hypercholesterolemic rabbits as they have more similar pathologic characteristics to human atherosclerosis than mice.

## Results

### Atherosclerotic Area and Aortic Lipids

**Figure 1:**
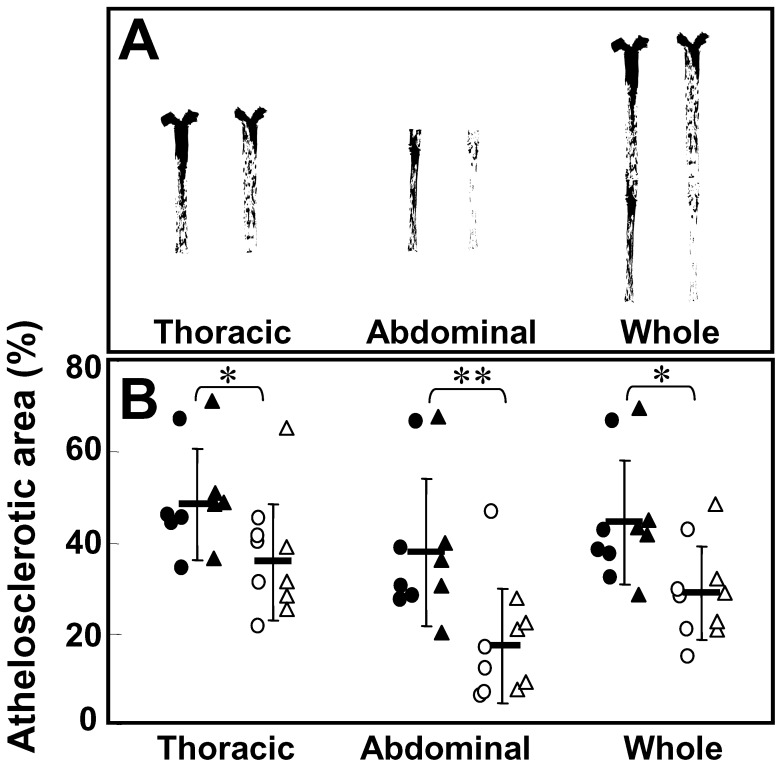
Comparison of atherosclerotic areas in aorta. Plaques areas in the aorta of a typical animal in each group are shown by black areas (A). Individual values in the control group are shown by closed triangles (previous study) and closed circles (present study) and those in the cilostazol group by opened triangles (previous study) and opened circles (present study) and the horizontal bars in each group represent the mean and the vertical bars are the SD (B). Control [n = 10], cilostazol [n = 10]. *: p<0.05, **: p<0.01 versus control by a two-way ANOVA.

**Figure 2:**
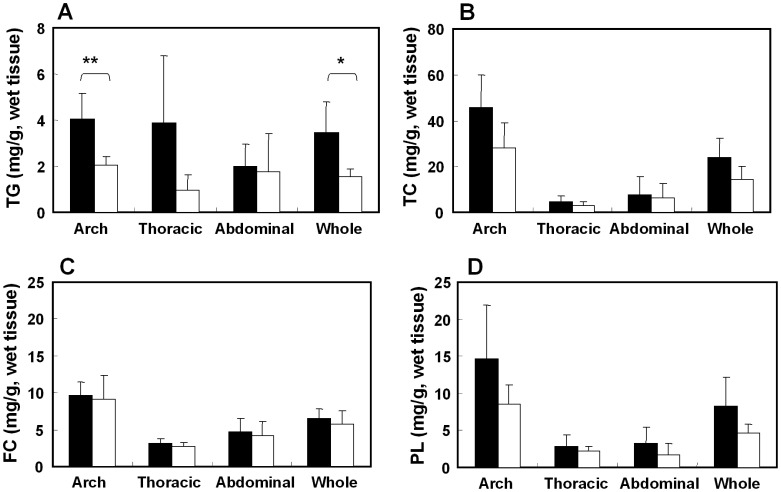
Effects of cilostazol on the lipid contents in the atherosclerotic aorta. Amounts of triglyceride (A), total cholesterol (B), free cholesterol (C) and phospholipid (D) in different regions of the aorta. Individual values of the control group are shown by closed columns and those of the cilostazol group by opened columns. Data represent means ± S.D. Control [n = 5], cilostazol [n = 5]. *: p<0.05, **: p<0.01 versus control by t-test.

The rate of the atherosclerotic area in the whole aorta in the control group was 44.4±13.6% and it was significantly attenuated by cilostazol treatment (28.8±10.3%, p = 0.0185) as shown in Figure 1. The reduction in atherosclerotic areas by cilostazol treatment was also significant in both the thoracic aorta (control : cilostazol, 48.4±12.2∶35.8±12.6%, p = 0.0443) and the abdominal aorta (control : cilostazol, 38.2±16.2∶17.6±12.6%, p = 0.0087). On the other hand, the triglyceride (TG) content in the cilostazol group was significantly lower than in the control group in the arch and the whole aorta (arch: 4.0±1.1 versus 2.0±0.4 mg/g, p = 0.006, whole: 3.5±1.3 versus 1.5±0.4 mg/g, p = 0.029) as shown in Figure 2. The total cholesterol (TC) contents in the cilostazol group tended to be lower than in the control group in the arch and the whole artery (arch: 45.8±14.2 versus 28.0±11.0 mg/g, p = 0.058, whole: 24.1±8.2 versus 14.4±5.7 mg/g, p = 0.062). The free cholesterol (FC) and phospholipid (PL) did not differ between the control and cilostazol groups in each region. The wet weights of the aortas showed no differences between the control group and cilostazol group.

### Intimal Macrophages

**Figure 3:**
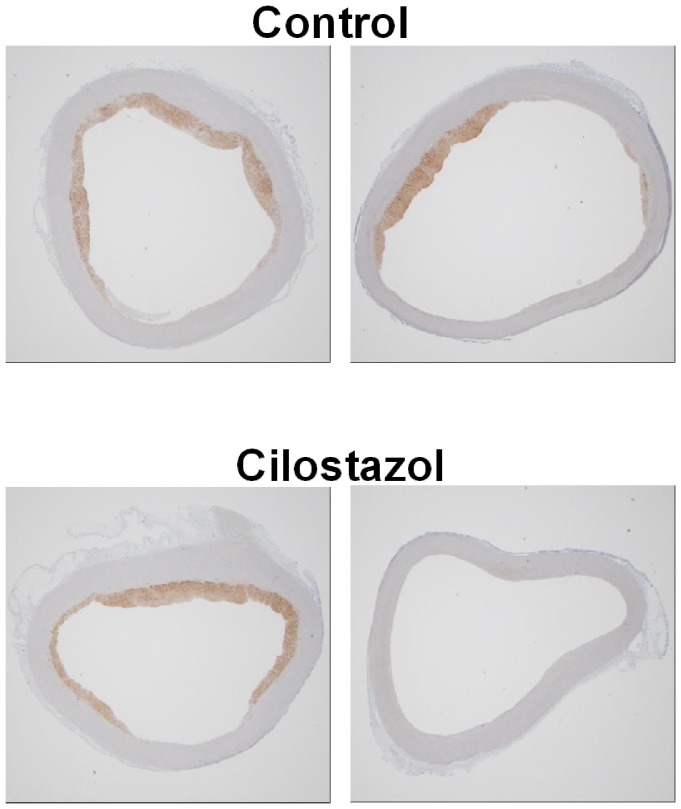
Immunohistochemical staining of macrophages in the proximal ascending aorta. Cross sections of the proximal ascending aorta were stained by an anti-macrophage RAM11 antibody. The brown part represents the macrophage-positive area. Typical images are shown for the control and cilostazol groups. Two of the five samples in the cilostazol group were not stained by the RAM11 antibody (the right image of cilostazol), although all samples were stained in the control group.

The results of immunohistochemical staining of macrophages in the proximal ascending aorta are shown in Figure 3. The mean values of the macrophage-positive areas did not differ between the control group (1.32±0.71 mm^2^) and the cilostazol group (1.46±1.33 mm^2^). However, two of the five samples in the cilostazol group did not have a macrophage-positive area, although all samples in the control group had macrophages. Additionally, the same section was stained by the Elastica-van Gieson method. The ratio of intima/media thickness in the aorta when measured at the same location did not differ between the two groups (data not shown).

### Platelet Aggregation

**Figure 4:**
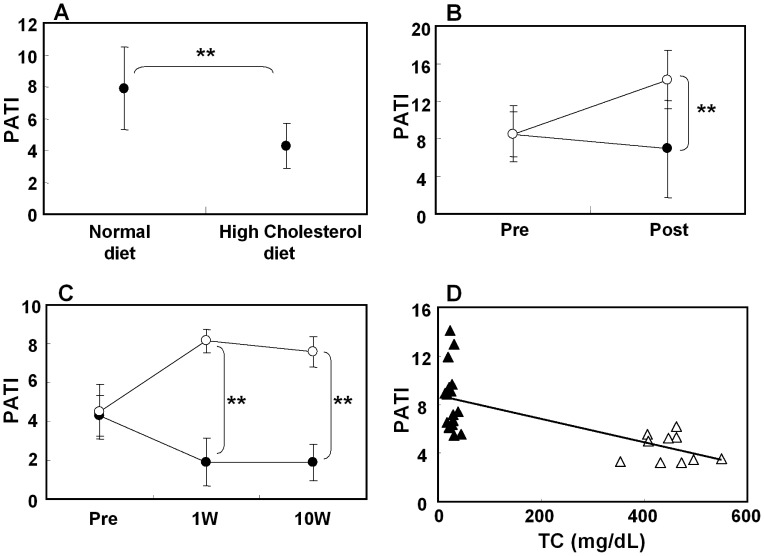
Platelet aggregation in rabbits. Comparison of platelet aggregation between normal and hypercholesterolemic rabbits (A). Effect of cilostazol on platelet aggregation in normal rabbits (B) or hypercholesterolemic rabbits (C). The control group are shown by closed circles and the cilostazol group by open circles. Correlation between serum TC and PATI values (D). Normal rabbits are shown by closed triangles and hypercholesterolemic rabbits by open triangles. Data represent means ± S.D. Normal rabbit study: control [n = 8], cilostazol [n = 8], hypercholesterolemic rabbit study: control [n = 5], cilostazol [n = 5]. **: p<0.01 versus control by t-test. PATI: Platelet aggregatory threshold index, TC: total cholesterol, R: correlative rate.

Platelet aggregability was expressed as a platelet aggregatory threshold index (PATI) value which is the concentration of agonist required to cause 50% platelet aggregation. Thus, if the PATI value for a certain condition is high, it means that platelet aggregability is suppressed under that condition. The PATI value for the high cholesterol diet was significantly lower than that in a normal diet indicating that platelet aggregability was enhanced by high-cholesterol feeding (Figure 4-A). When rabbits were fed a normal diet, cilostazol significantly inhibited the platelet aggregation 1 week after drug treatment (Figure 4-B). Furthermore, cilostazol significantly prevented the platelet aggregation 1 week after drug treatment in rabbits fed a 0.5% cholesterol diet (Figure 4-C). The inhibitory effect of cilostazol was maintained 10 weeks after drug treatment. A significant linear correlation was observed between the serum TC concentrations and the PATI values prior to drug treatment (Figure 4-D, correlation coefficient: r = –0.6897, p<0.001).

### Serum Lipids and Other Biomarkers

**Figure 5:**
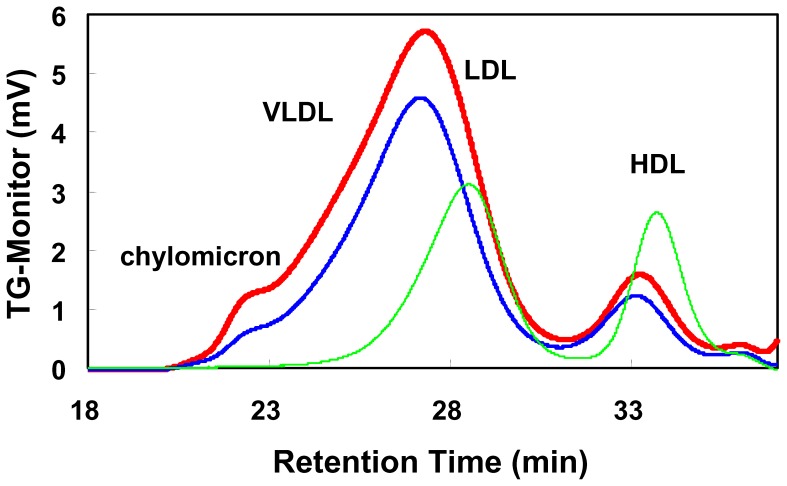
Serum TG analysis by HPLC. Lipoproteins in 10 week treatment serum samples were analyzed by HPLC. The mean value of the control group is shown by a red line and the cilostazol group by the blue line and the normal diet group by the green line. Control [n = 5], cilostazol [n = 5], normal diet [n = 3].

Changes in serum lipids and other biomarkers are shown in Table 1. Serum TC, TG and PL levels were gradually elevated until the 10th week in the control group fed the 0.5% cholesterol diet. The elevation of the serum lipids was significantly reduced by cilostazol treatment; TC: p = 0.0435, TG: p = 0.0105 and PL: p = 0.0144 (group*time interaction in repeated measures analysis of variances). Serum FC also increased in the control group and tended to be suppressed by cilostazol treatment (p = 0.1169, group*time). Serum HDL-C did not show any change in the control but increased in the cilostazol group from the 2nd week to the 4th week (p = 0.0239, group*time, p = 0.0361, group difference). Using serum glutamate oxaloacetate transaminase (GOT), glutamate pyruvate transaminase (GPT) and alkaline phosphatase (ALP) as indices of hepatic function no differences between the control and cilostazol groups were noted. Serum C-reactive protein (CRP), an index of inflammation, markedly increased until the 10th week although the CRP levels contained considerable individual variability. Cilostazol did not affect the elevation of serum CRP. By serum TG monitoring by HPLC, the high fat diet increased TG in very low-density lipoprotein and chylomicron fractions, and cilostazol reduced TG in the same fractions (Figure 5).

**Table 1:** Changes of serum lipids and biomarkers.

	Group	Pre	2W	4W	6W	8W	10W	
**TC**	Control	475±44	1109±133	1492±148	1781±143	2147±491	2398±379	
	Cilostazol	422±53	963±269	1140±449	1283±627	1466±579	1618±705	*
**TG**	Control	21±8	37±13	60±25	110±63	523±26	569±55	
	Cilostazol	21±3	26±11	47±24	59±41	445±71	470±68	*
**FC**	Control	105±9	236±37	539±72	359±59	520±101	596±124	
	Cilostazol	98±12	219±40	432±168	262±135	359±182	393±172	
**HDLC**	Control	22±4	31±6	32±8	34±4	28±4	30±6	
	Cilostazol	22±5	38±7	40±7	34±5	31±4	25±7	*, †
**PL**	Control	183±14	352±28	442±30	525±48	630±56	764±198	
	Cilostazol	181±25	344±71	411±136	420±166	474±180	520±213	*
**GOT**	Control	23±12	33±33	31±18	27±8	30±9	45±43	
	Cilostazol	32±30	32±25	30±14	26±11	28±11	25±9	
**GPT**	Control	38±13	45±24	52±26	40±12	44±8	56±31	
	Cilostazol	94±100	75±59	80±51	66±42	61±30	72±45	
**ALP**	Control	654±178	401±96	486±137	412±59	364±78	374±91	
	Cilostazol	663±303	336±155	462±177	384±127	359±129	431±171	
**CRP**	Control	955±346	7843±3115	4167±4111	13418±15039	11164±10216	93760±186537	
	Cilostazol	1665±1119	9653±4829	6097±4856	17544±19860	9965±7674	23683±21739	

Concentrations of serum lipids and biomarkers were measured before and 2, 4, 6, 8, 10 weeks after drug administration. Data represent means ± S.D. Control [n = 5], cilostazol [n = 5]. *: p<0.05 (group*time), †: p<0.05 (group) versus control by repeated measures ANOVA. TC: total cholesterol, TG: triglyceride, FC: free cholesterol, HDL-C: high density lipoprotein cholesterol, PL: phospholipid, GOT: glutamate oxaloacetate transaminase, GPT: glutamate pyruvate transaminase, ALP: alkaline phosphatase, and CRP: C-reactive protein. TC, TG, FC, HDL, PL is shown at mg/dL, GOT, GPT, ALP at U/L and CRP at ng/mL.

### Serum Concentration of Cilostazol

Each morning, blood samples were collected to measure the cilostazol concentration in serum 1 hour after feeding. The serum concentrations of cilostazol were 256±86 ng/mL from rabbits fed a normal diet and 294±198 ng/mL from rabbits fed a high-cholesterol diet, approximately one-third of that normally obtained in the clinic.

## Discussion

The present study is the first to evaluate the effects of cilostazol on atherosclerosis and ex vivo platelet aggregation in hypercholesterolemic rabbits. We showed that cilostazol significantly reduced the atherosclerotic area and the serum TG and increased the serum HDL-C in addition to reducing TC and PL in hypercholesterolemic rabbits. Additionally, it is particularly worth noting that cilostazol significantly reduced the aortic TG in the rabbits although aortic lipids were not measured in the previous studies of atherosclerotic mice [13]–[15]. Cilostazol strongly reduced serum triglycerides with a slight increase in HDL-cholesterol after 2 and 4 weeks of treatment. The results agree well with the results from patients with peripheral arterial disease (PAD) [18]–[20]. Cilostazol may be more effective at reducing serum TG than increasing the HDL-C. Others have also reported that cilostazol reduced pro-atherogenic lipoproteins such as remnants-like particles, which is a strong contributor to cardiovascular risk, in the plasma of patients with PAD and/or type 2 diabetes [18]–[22]. Hirano et al. has reported that TG deposits were found in the cardiac arteries of a patient with cardiomyopathy [25], and the reduction of aortic TG content has clinical implication. Maeda et al. studied the effect of cilostazol on the TG secretion from the liver using an inhibitor of lipoprotein lipase in rats, and found that the rate of TG secretion was decreased by cilostazol [17]. On the other hand, Tani et al. have demonstrated that cilostazol increased lipoprotein lipase activity, resulting in a decrease in serum TG levels in streptozotocin-induced diabetic rats [16]. These cilostazol-induced actions might contribute to the reduction of serum TG in hypercholesterolemic rabbits. It is also known that pharmacological agents that increase intracellular cyclic AMP levels can enhance HDL_3_-mediated, sterol efflux from cholesterol-loaded human skin fibroblasts and bovine aortic endothelial cells [26]. The mild elevation of HDL-C by cilostazol treatment noted in this study might be due to the same mode of action because cilostazol increases intracellular cyclic AMP levels by inhibiting phosphodiesterase 3. It is well-known that a high level of low-density lipoprotein cholesterol and a low level of HDL-C are principal risk factors for coronary heart disease and stroke [27]–[28]. Serum TG is also one of the principal risk factors for arterial thrombotic diseases [29]–[30]. Decreasing serum and aortic TG by cilostazol treatment may contribute to the prevention of the atherothrombotic diseases.

In contrast to the significant reduction in triglyceride content in the aorta, treatment with cilostazol did not show a statistically significant effect on the atherosclerotic area. We have investigated the anti-atherosclerotic effect of cilostazol by treatment in a dose-dependent manner (0.1, 0.3 and 1.0% cilostazol diet) in cholesterol-fed rabbits ahead of the present study, and we observed that a 0.3% cilostazol diet tended to reduce the atherosclerotic area. Therefore, in this study, we confirmed the reproducibility of the anti-atherosclerotic effect of cilostazol and examined in more detail the effects of cilostazol on serum lipids, aorta lipids, and platelet aggregation. The anti-atherosclerotic efficacy of cilostazol was analyzed using the combined results from the dual study. A significant linear correlation was observed between serum TG and aortic TG (correlation coefficient: r = 0.5745) but no correlation between serum CRP and aortic TG was noted. In addition, from the result obtained for the serum TG monitoring by HPLC, it was speculated that the reduction of triglyceride in both very low-density lipoprotein and chylomicron by cilostazol might contribute to the reduction of triglyceride accumulation in the aorta. Clarification of the mechanism of TG reduction by cilostazol requires further study. The evaluation of the atherosclerotic area is meaningful to detect whether the plaque is abundant or not. Actually, more plaques were observed on the thoracic aorta than on the abdominal aorta and the plaques were abundant around the arterial bifurcation. It is possible that irregular local shear stress might be involved in the plaque formation at such areas. The local shear stress is one of the important factors affecting platelet functions. Cilostazol inhibited the shear stress-induced platelet aggregation [31].

From the immunostaining analysis of the macrophages in the proximal ascending aorta, there was no difference between the macrophage-positive areas in the two groups. However, it may be a notable result that 2 of the 5 samples in the cilostazol group were not stained by the anti-macrophage antibody, while all samples were stained in the control group. Cilostazol inhibited the monocyte adhesion to endothelia activated by remnant lipoprotein [32]. The results in this study might partially reflect the inhibitory effect of cilostazol on the monocyte adhesion. However, further study is warranted to elucidate the exact effect of cilostazol on macrophage accumulation in atherosclerotic plaques.

It has recently been reported that cilostazol inhibited the uptake of modified low-density lipoprotein cholesterol and foam cell formation in mouse peritoneal macrophages by reduction in the expression of scavenger receptors [33]. Moreover, cilostazol has been shown to inhibit the proliferation of smooth muscle cells and protect from endothelial injury [34]–[35]. Monocyte adhesions to the endothelium and platelets are also prevented by cilostazol [36]–[37]. Thus, the pleiotropic effects of cilostazol, such as its anti-platelet, anti-atherosclerotic and anti-inflammatory actions may be important for preventing the progression of atherosclerotic disease.

The light transmission method has been generally used for the measurement of platelet aggregation. In this conventional method, the change in light transmission following addition of an agonist is traced as a parameter of platelet aggregation, where the light transmission of the platelet-poor plasma is set at 100% and that of the platelet-rich plasma as 0%. Therefore, the method is unsuitable for measuring platelet aggregation when the plasma is clouded due to hyperlipidemia. Thus, in the present study, we measured platelet aggregation using a SFP aggregometer as it is not affected by the turbidity of plasma. The method is also easy as the preparation of platelets by centrifugation is unnecessary. Additionally, generally results obtained using the SFP method correlate well to the results obtained by the light transmission method [5].

During the development of atherosclerosis, the interaction between platelets and the vascular wall is critical. When the vascular walls are damaged, platelets adhere to the subendothelial surface containing collagen fibers and aggregate [1], [38]. Platelet sensitivity is enhanced with hypercholesterolemia in rabbits and human [2]–[3]. Son et al. reported that the platelet aggregation in the hypercholesterolemic rabbit was induced by a low concentration of collagen [3]. In the present study, platelet aggregability in hypercholesterolemic rabbits was also enhanced, and this hyper-reactivity was maintained for up to 10 weeks when fed a 0.5% cholesterol diet. The correlation between the serum TC concentration and the PATI was significant; the higher the serum TC, the lower the PATI. It is known that lowering of the serum TC attenuates platelet aggregation as well as atheroma formation [39]. Cilostazol inhibits platelet aggregation by various agonists, in particular, it strongly inhibits platelet aggregation induced by collagen [11], one of the major components of the subendothelium. The inhibitory effect of cilostazol on platelet aggregation was maintained throughout the duration of this study. Thus, the anti-atherosclerotic effect of cilostazol might partly result from its sustained anti-platelet effect.

In summary, cilostazol improved lipid levels in serum and atherosclerotic aorta and inhibited the platelet aggregation detected using a SFP whole blood aggregometer in hypercholesterolemic rabbits. Cilostazol may be useful to prevent atherosclerotic progression through its anti-platelet effect and help to improve lipid abnormalities in hypercholesterolemic patients.

## Materials and Methods

### Experimental Animals

Seven-week-old male rabbits (New Zealand White) were purchased from Kitayama Labes (Nagano, Japan). To minimize the number of the animals used, the rabbits were washed out for 2 weeks between the first platelet aggregation study with a normal cholesterol diet and the second aggregation study with 0.5% cholesterol diet. Rabbits were fed each particular diet at 100 g/day/animal. All experimental procedures were performed in accordance with the Guidelines for Animal Care and Use of Otsuka Pharmaceutical Co., Ltd. The ethics committee specifically approved this study.

### Design of Experiments

We first evaluated the platelet aggregation in rabbits fed a normal diet in the presence or absence of cilostazol, and then all the rabbits were fed the normal diet for 2 weeks to wash out the drug. Next, the platelet aggregation was evaluated in rabbits fed a high cholesterol diet. Finally, we evaluated the atherosclerotic area in aorta and the lipids in the serum and aorta. Using the combined results from the dual study, the evaluation of the atherosclerotic area was carried out.

### Materials

The SFP whole blood aggregometer was from Mebanix Co., Ltd. (WBA analyzer, Yokohama, Japan). Cilostazol was synthesized at Otsuka Pharmaceutical Co., Ltd. Collagen was purchased from Nycomed Arzneimittel Gmbh. (Munchen, Germany) and sodium citrate from Sysmex (Kobe, Japan).

### Measurement of Platelet Aggregation by SFP Aggregometer

Measurement of platelet aggregation with the SFP aggregometer was carried out according to the method previously described [7]. Briefly, blood was collected from animals into plastic syringes containing sodium citrate at a final concentration of 0.38%. Suspensions of collagen were prepared at 10 times the final concentrations at 4°C. Four reaction tubes containing 200 µL aliquots of whole blood with a stirring bar were placed in the incubation chamber at 37°C. The reaction tubes were pre-incubated for 1 min at 37°C, and then 22.2 µL each of four concentrations of collagen were added. Five minutes thereafter, the filter-unit syringe with a screen micro-sieve was used to suck the blood samples. A pressure sensor was connected to the syringe. A negative pressure of –130 mmHg was set as 100% and 0 mmHg as 0%. The platelet aggregation of each reaction tube was determined as the pressure rate (%). The concentration of agonist causing a 50% change of pressure rate was calculated and expressed as the platelet aggregatory threshold index (PATI).

### Evaluation of Cilostazol on Platelet Aggregation

We firstly evaluated the platelet aggregation in rabbits fed a normal diet. Platelet aggregation was measured in twenty rabbits and the PATI (pre) before drug treatment was calculated for each animal. The rabbits were then divided into two groups (control group and cilostazol group) with eight animals per group, and four animals were excluded because of an outlier PATI. The PATI (post) was measured 1 week after drug treatment, then, all the rabbits were fed the normal diet for 2 weeks to wash out the drug. Next, the platelet aggregation was evaluated in rabbits fed a 0.5% cholesterol diet. The total cholesterol in serum was measured before and after feeding the 0.5% cholesterol diet for a week. After feeding the 0.5% cholesterol diet for 1 week, platelet aggregation was determined as the PATI (pre) for the high cholesterol diet before drug treatment. Ten rabbits were excluded since the serum TC level was too high (>700 mg/dL) or too low (<300 mg/dL) or because of the outlier PATI, and the remaining animals were allocated into two groups (control group and cilostazol group) with five animals per group. A 0.5% cholesterol diet and 0.5% cholesterol plus 0.3% cilostazol diet were fed to the control group and the cilostazol group, respectively for 10 weeks. The platelet aggregation was measured and the PATI (post) 1 week and 10 weeks after drug treatment was calculated for the rabbits fed a high cholesterol diet.

### Measurement of Serum Lipids and other Biomarkers

For the rabbits fed the high cholesterol diet, the blood was collected before and 2, 4, 6, 8 and 10 weeks after drug treatment, and the serum was obtained by centrifuging it at 2000×g for 10 minutes at room temperature. Total cholesterol, TG, FC, PL and HDL-C were measured as serum lipids, and GOT, GPT and ALP were measured as indices of hepatic functions and CRP was used as an index of inflammation. Serum lipids excluding FC, and GOT, GPT and ALP were measured by the multi-function automatic analyzer system (BiOLis 24i, Tokyo Boeki Machinery Ltd, Tokyo, Japan). Serum FC was measured using a multi-plate reader (Soft MAX Pro, Spectra MAX 190, Molecular Devices, CA, USA) using reagents for the BiOLis 24i. Serum CRP was measured by an enzyme-linked immunosorbent assay kit (Shibayagi, Gunma, Japan). Lipoproteins were analyzed by HPLC from 10 week serum samples. Serum was fractionated by gel chromatography using a column for lipoprotein analyses (TSKgel LipopropakXL, Tosoh, Japan). After gel filtration, lipoprotein was colored with TG reaction liquid in reaction coils, and the absorbance at 600 nm was monitored, and the elution pattern was recorded.

### Measurement of Atherosclerotic Areas

The analysis of the atherosclerotic lesion was performed by Daugherty’s method with a slight modification [40]. Following the last platelet aggregation study, rabbits were anesthetized by an injection of pentobarbital sodium (40 mg/mL at volume of 1 mL/kg animal) into the ear vein and were held on an experimental plate and their body hair was shaved. The abdomen and chest were opened, and then blood (60 mL) was collected from the abdominal vein. The aorta was perfused with ice-cold saline. The length of the aorta from the origin of the aortic arch to a femoral artery bifurcation was measured, and the aorta was excised and placed in cold saline. The aorta was cut into two regions at the diaphragm, i.e. the thoracic aorta including the arch and the abdominal aorta, and was trimmed to remove extraneous tissues under the stereomicroscope (SMZ800, Nikon, Tokyo, Japan), then longitudinally incised, and the intimal sides were exposed. The aortas were pinned on black rubber plates and photographed with a digital camera (D80, Nikon, Tokyo, Japan). The whole intimal and atherosclerotic areas were analyzed using image analyzers (Photoshop, Adobe Systems, Tokyo, Japan and WinROOF, Mitani Corporation, Tokyo, Japan). The ratio of the atherosclerotic area to the whole intimal area was calculated. Plaque areas of unstained aortas were measured in the present study because the plaque areas strongly correlated with the Sudan IV staining areas in the preliminary study (correlation coefficient: r = 0.9898).

### Measurement of Aortic Lipids

After obtaining the photograph, the thoracic aorta was further separated into the arch and the residual thoracic aorta. The wet weights of the aortas (arch, thoracic aorta, and abdominal aorta) were weighed by an electronic balance (CP225D, Sartorius, Tokyo, Japan). The aortas were chopped into fine pieces. The extraction liquid (chloroform/methanol (2∶1, v/v)) was added to the chopped arteries, and they were homogenized by a digital homogenizer (As-one, Osaka, Japan). The homogenates were centrifuged at 800×g for 10 minutes. The upper-layer solution was transferred into another tube and mixed with 0.36 M CaCl2/50% methanol. The mixture was centrifuged at 800×g for 10 minutes, and this washing procedure was repeated twice. The 100 µL aliquot of the solution used to extract lipids was dried with 50% Triton X-100 in chloroform on a dry-block bath (As-one, Osaka, Japan). Isopropanol was added to the dried sample. Lipids (TC, TG, FC and PL) in the samples were measured as serum lipids.

### Histochemical Staining of the Aortic Cross Section

Proximal ascending aortas were fixed with 10% neutral buffered formalin, embedded in paraffin, sectioned at 3 µm and adhered to APS coated slides. Sections were stained with an automatic immunohistochemical staining system (Venatana HX system Discovery, Roche-Diagnostics) using a mouse monoclonal antibody against rabbit macrophage, RAM11 (1∶200, Dako). Additionally, the same section was stained by the Elastica-van Gieson method.

### Statistical Analysis

For the PATI values, lipids in the aorta and the rate of RAM11 positive cells, the differences between the control group and the cilostazol group were statistically analyzed by a t-test. For serum lipids, the differences between two groups were statistically analyzed by repeated measures analysis of variance (ANOVA). The rate of the atherosclerotic area was statistically analyzed by using two-way ANOVA. Factors included in the ANOVA model were group and time interaction. The correlations between the serum TC concentrations and PATI values, the serum TC and serum TG, the plaque areas and the Sudan IV staining areas were evaluated using Pearson’s correlation coefficient and correlation coefficient test. Values were expressed as means ± S.D. A p-value <0.05 was considered significant. Analyses were performed using the SAS software (release 9.1, SAS Institute Japan, Tokyo, Japan).
